# Supplementary material for: Mapping multi-dimensional variability in water stress strategies across temperate forests
Source: Nat Commun. 2024 Oct 16;15:8909. doi: 10.1038/s41467-024-53160-1 (PMC11484845; doi:10.1038/s41467-024-53160-1)
Supplement: Supplementary file 1 — Supplementary Information [file 41467_2024_53160_MOESM1_ESM.pdf]

Supplementary Information

Mapping multi-dimensional variability in water stress strategies across temperate forests

Daijun Liu[1,2,3]; Adriane Esquivel-Muelbert[1,2]; Nezha Acil[1,2,4,5]; Julen Astigarraga[6]; Emil Cienciala[7,8], Jonas Fridman[9]; Georges Kunstler[10]; Thomas J. Matthews[1,2,11]; Paloma Ruiz-Benito[6,12]; Jonathan P. Sadler[1,2]; Mart-Jan Schelhaas[13]; Susanne Suvanto[1,2,14]; Andrzej Talarczyk[15]; Christopher W. Woodall[16]; Miguel Zavala[6]; Chao Zhang[17]; Thomas A. M. Pugh[18,1,2]

Figures

Figure S1. The histogram plot for the number of forest plots in each community in our study.

Figure S2. Key functional strategies along the two dimensions for the woody species across regions.

Figure S3. Distributions of trait observations at species level and community level.

Figure S4. Differences in functional strategies among the functional groups along PC1 and PC2.

Figure S5. The predicted trait values associate with the original values.

Figure S6. The PCA results for the species level traits with respect to the three methods for filling the trait gaps.

Figure S7. The PCA results for the community level traits with respect to different sizes of grid cells and to methods for filling the trait gaps.

Figure S8. Procrustes tests for the comparison among three different gap-filling methods for the traits at species level.

Figure S9. Procrustes tests for the comparison among three different gap-filling methods for the forest communities with different grid sizes.

Figure S10. Sensitivity test for key functional strategies along the two dimensions for the forest communities larger than 2 plots.

Figure S11. Geographic distribution of the dominant plant functional types across USA and Europe.

Figure S12. Comparing the mean trait variance within and forest communities.

Tables

Table S1. Forest inventory data used in our paper.

Table S2. Percentage of traits missing for the woody species in our analyse.

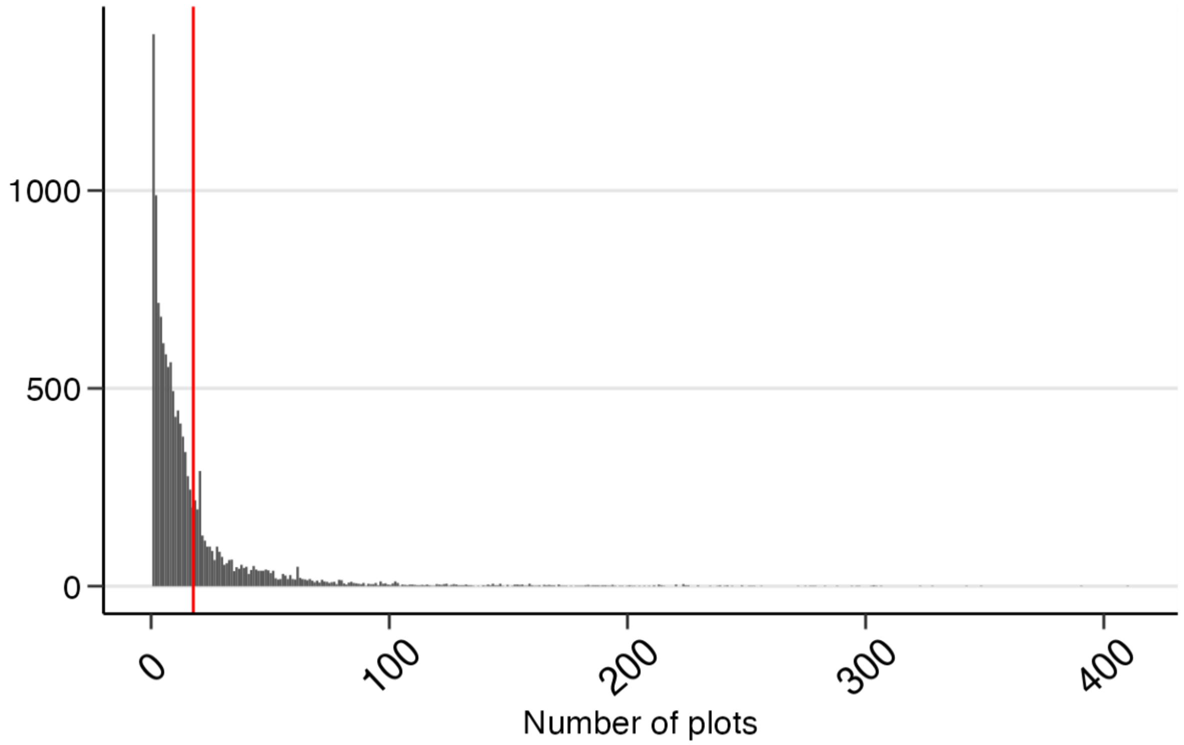

Figure S1. The histogram plot for the number of forest plots in each community in our study. The red line is the median forest plot number (n=17.3). There are 12,452 forest communities (0.25° grid cell) across the regions of the USA and Europe (i.e., Spain, France, Germany, Czechia, Poland and Sweden).

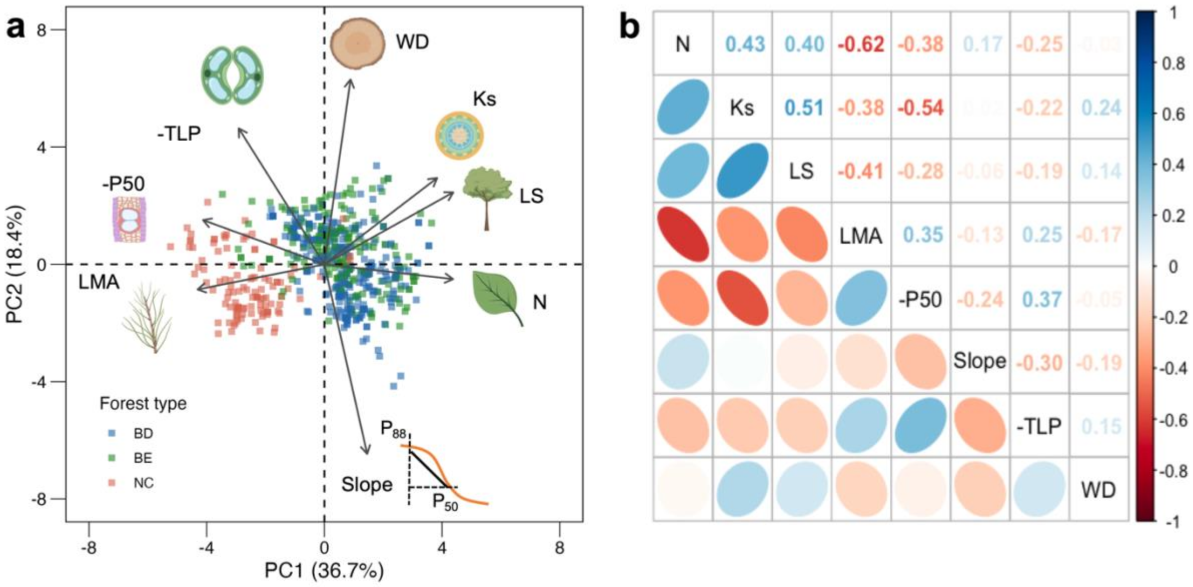

Figure S2. Key functional strategies along the two dimensions for the woody species across regions. a. Biplot resulting from the principal component analysis (PCA) of eight functional traits at species level. The dominant forest type was identified according to their leaf phenological types: BD (broadleaved deciduous, blue), BE (broadleaved evergreen, green) and NC (needleleaved conifer, red)). There are 643 woody species used in the analysis, with 307, 215 and 121 used for BD, BE and NC; respectively. b. Pearson correlation coefficient (p values <0.001) between the functional traits at species level is shown. Abbreviations of functional traits: N (leaf nitrogen content); Ks (maximum xylem conductivity per unit sapwood area); LS (leaf area to sapwood area ratio); LMA (leaf mass per area); P50 (xylem water potential at 50% loss of conductivity); Slope (slope for the embolism vulnerability curve between P50-P88); TLP (leaf turgor loss point) and WD (Wood density). The detailed traits and explanations are shown in Table 1. The elements in *a* are created in BioRender. Zhang, C. (2024) BioRender.com/u64o243.

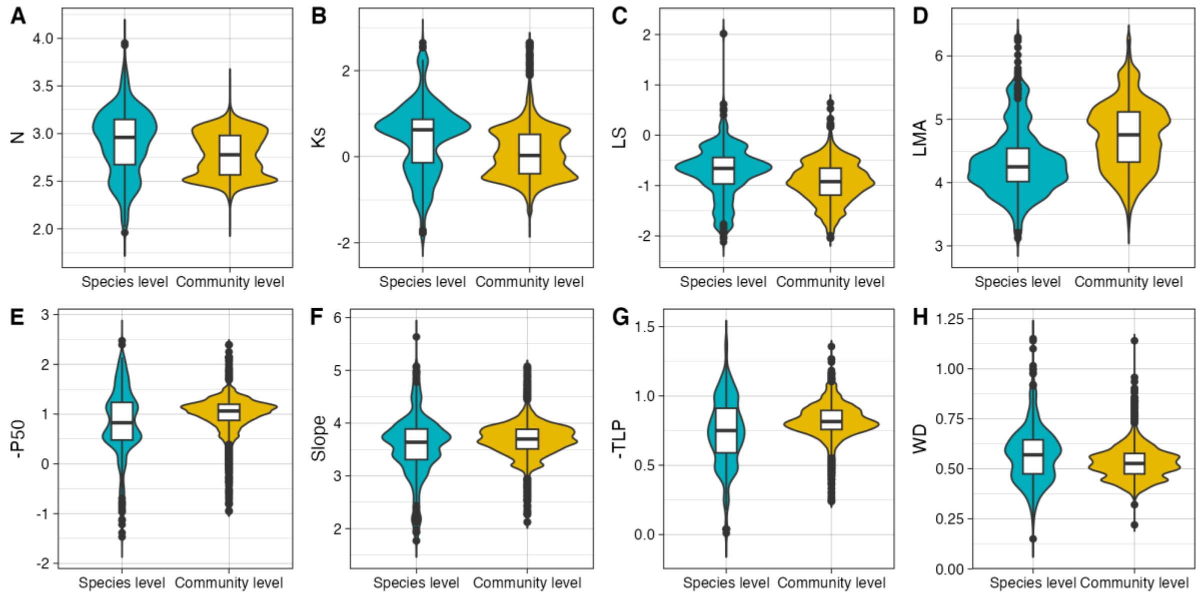

Figure S3. Distributions of trait observations at species level and community level. Eight traits are used in our analysis. There are 643 species and 12,452 forest communities used into the analysis at the species level and community level; respectively. Violin plots show the probability density of the trait values at the species-level and community-level separately. The boxplots indicate the interquartile range (the first quartile (Q1) and the third quartile (Q3)), which includes the median (50th percentile) of the data. The whiskers represent the minimum and maximum values within 1.5 times the interquartile range from Q1 and Q3, respectively. Outliers are trait values over 1.5 times the interquartile range over the 75th percentile or fall below 1.5 times the interquartile range under the 25th percentile. Abbreviations of functional traits: N (leaf nitrogen content); Ks (maximum xylem conductivity per unit sapwood area); LS (leaf area to sapwood area ratio); LMA (leaf mass per area); P50 (xylem water potential at 50% loss of conductivity); Slope (slope for the embolism vulnerability curve between P50-P88); TLP (leaf turgor loss point) and WD (Wood density). The detailed traits and explanations are shown in Table 1.

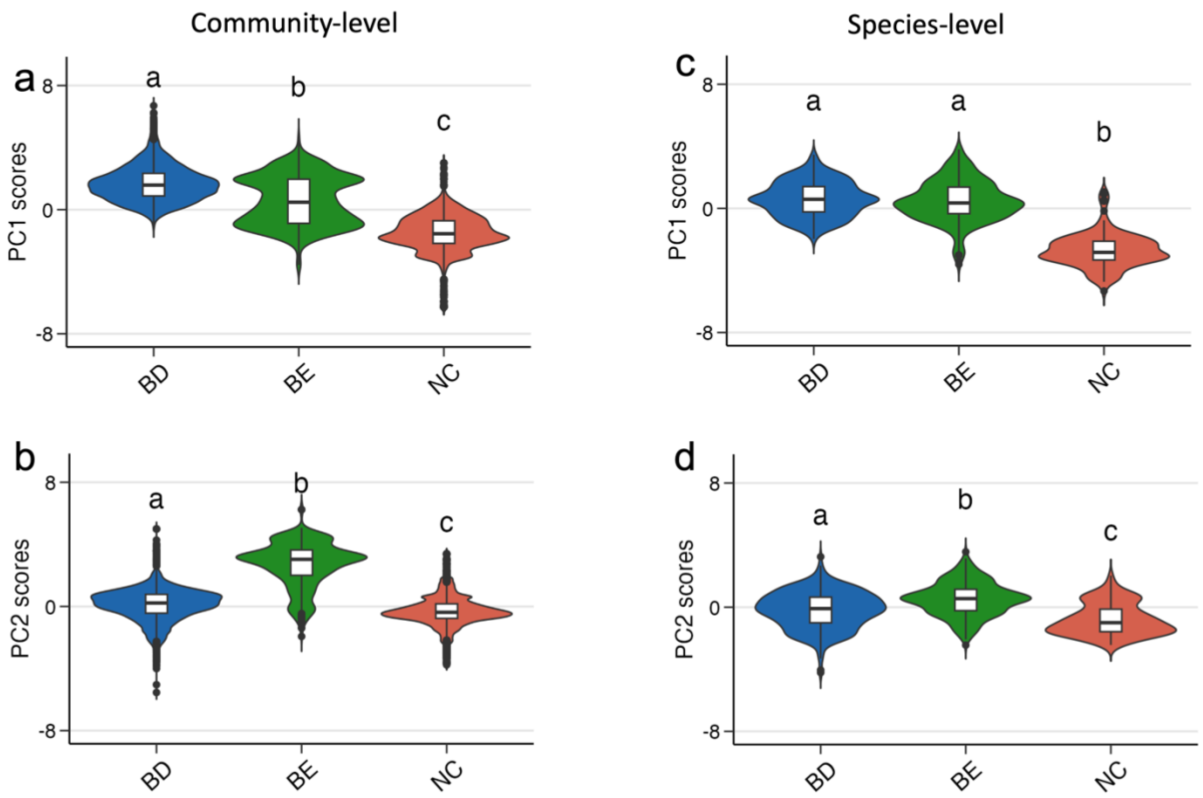

Figure S4. Differences in trait associations among the functional groups along PC1 and PC2. We applied a one-way analysis of variance (ANOVA) with Tukey's HSD (honest significant difference) post-hoc test to test the difference in strategies among different groups. Different letters indicate the significance among the main plant functional types at community level (a, b) and species level (c, d). The different colours indicate plant functional types (PFTs): broadleaved deciduous (BD, blue), broadleaved evergreen (BE, evergreen) and needleleaved conifer (NC, red). There are 5,584 BD, 407 BE and 6,461 NC at community level and 307 BD, 215 BE and 121 NC at species level. Violin plots show the probability density of the trait values. The boxplots indicate the interquartile range (the first quartile (Q1) and the third quartile (Q3)), which includes the median (50th percentile) of the data. The whiskers represent the minimum and maximum values within 1.5 times the interquartile range from Q1 and Q3, respectively. Outliers are trait values over 1.5 times the interquartile range over the 75th percentile or fall below 1.5 times the interquartile range under the 25th percentile.

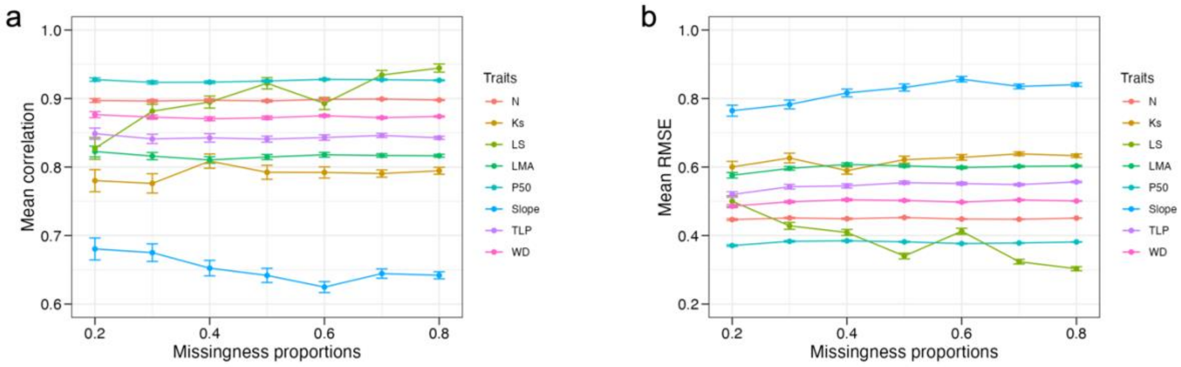

Figure S5. Effectiveness of the gap-filling methods for species trait values. We randomly selected different proportions of missingness (0.2, 0.3, 0.4, 0.5, 0.6, 0.7 and 0.8) of these species which we have observations of trait values and predicted their values from the remaining of the species, following the gap-filling method - the median of genus and family and phylogenetic relationship (default, as described in the main text). We then randomly predicted 100 times to calculate the correlation coefficient (R^2) and root mean squared error (RMSE) of z-transformed predicted versus observation trait values were used as indicator of overall prediction accuracy for trait gap-filling. The mean values and standard errors for the R^2 (Fig. S5a) and RMSE (Fig. S5b) were shown; respectively. Abbreviations of functional traits: N (leaf nitrogen content); Ks (maximum xylem conductivity per unit sapwood area); LS (leaf area to sapwood area ratio); LMA (leaf mass per area); P50 (xylem water potential at 50% loss of conductivity); Slope (slope for the embolism vulnerability curve between P50-P88); TLP (leaf turgor loss point) and WD (Wood density). The detailed traits and explanations are shown in Table 1.

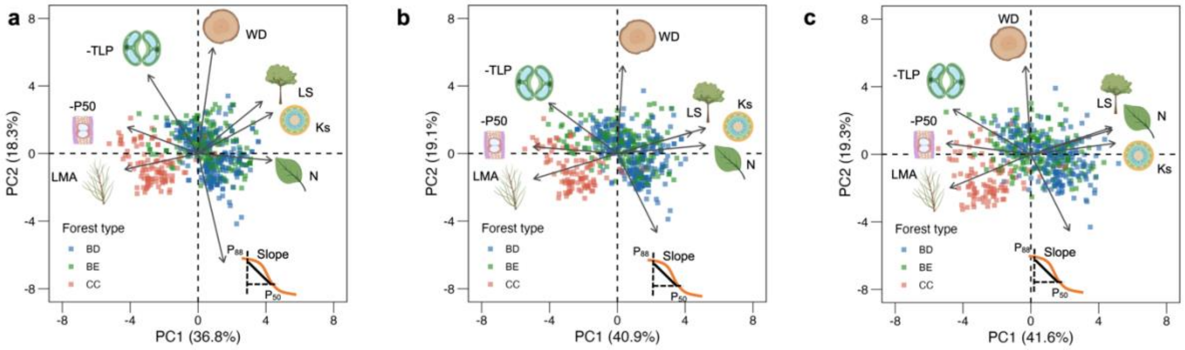

Figure S6. The PCA results for the species-level traits with respect to the three methods for filling the trait gaps. Three different methods were used to fill the missing trait gaps by applying (m1) the median of genus and family and phylogenetic relationship (a), (m2) median of genus and phylogenetic relationship (b) and (m3) only the phylogenetic relationship (c). The leaf type was identified according to their leaf phenological types: BD (broadleaved deciduous, blue), BE (broadleaved evergreen, green) and NC (needleleaved conifer, red)). The points with different colors indicate the groups of woody species. There are 643 woody species used in the analysis, with 307, 215 and 121 used for BD, BE and NC; respectively. Abbreviations of functional traits: N (leaf nitrogen content); Ks (maximum xylem conductivity per unit sapwood area); LS (leaf area to sapwood area ratio); LMA (leaf mass per area); P50 (xylem water potential at 50% loss of conductivity); Slope (slope for the embolism vulnerability curve between P50-P88); TLP (leaf turgor loss point) and WD (Wood density). The detailed traits and explanations are shown in Table 1. The elements in a-c are created in BioRender. Zhang, C. (2024) BioRender.com/u64o243.

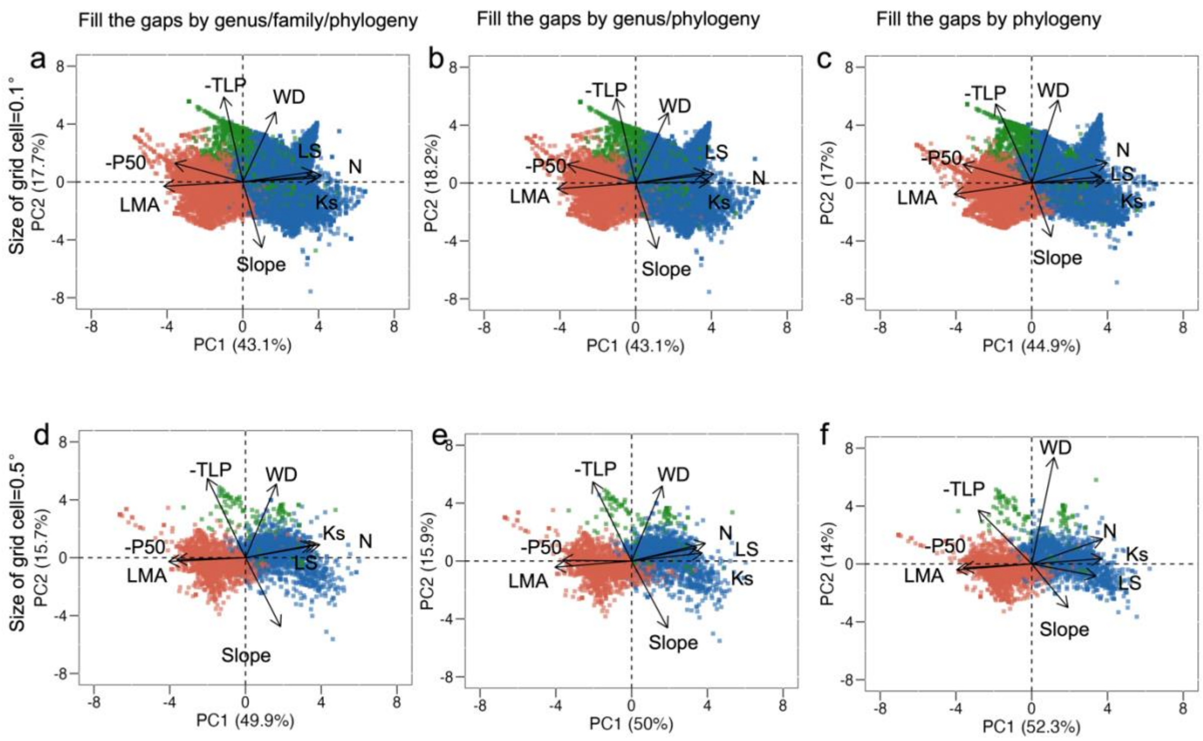

Figure S7. The PCA results for the community-level trait means with respect to different sizes of grid cells and to methods for filling the trait gaps. The forests plots have been aggregated into the sizes of 0.1° and 0.5°, respectively. And then eight key functional traits of woody species at species level have been assigned to the species in each community. The missing trait gaps have been filled by three methods. The PCA results of the community level traits of the grid cell size of 0.1° (a-c) and 0.5 ° (d-e) that the trait gaps were filled by three different methods in the sensitivity analysis (details in the Methods and Materials). The different colours indicate forest main functional types: broadleaved deciduous (BD, blue), broadleaved evergreen (BE, evergreen) and needleleaved conifer (NC, red). There are 49,805 and 3,798 forest communities for the 0.1° and 0.5° sizes, respectively. Abbreviations of functional traits: N (leaf nitrogen content); Ks (maximum xylem conductivity per unit sapwood area); LS (leaf area to sapwood area ratio); LMA (leaf mass per area); P50 (xylem water potential at 50% loss of conductivity); Slope (slope for the embolism vulnerability curve between P50-P88); TLP (leaf turgor loss point) and WD (Wood density). The detailed traits and explanations are shown in Table 1.

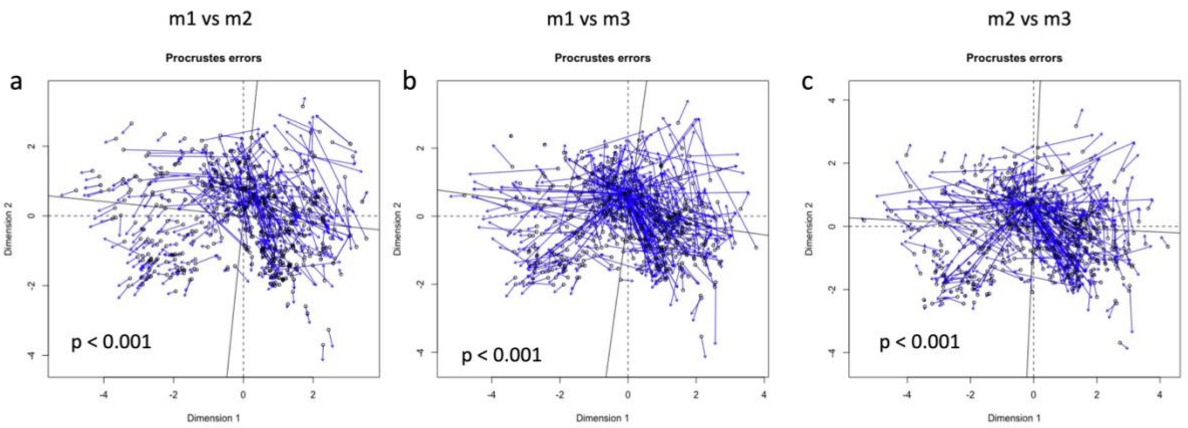

Figure S8. Procrustes tests for the comparison among three different gap-filling methods for the traits at species level. There are 643 woody species used in the analysis, with 307, 215 and 121 used for BD, BE and NC; respectively. Three different methods were used to fill the missing trait gaps by applying (m1) the median of genus and family and phylogenetic relationship, (m2) median of genus and phylogenetic relationship and (m3) only the phylogenetic relationship. The loading values of the two main dimensions of the PCA tests were used for the comparisons (m1 vs m2 (a); m1 vs m3 (b) and m2 vs m3 (c). a-c, The visual indication of the degree of match between the two ordinations. The arrows point to their positions in the target ordination. The plot also shows the rotation between the two ordinations necessary to make them match as closely as possible. The p values (all $p<0.001$) indicate that there were no significant differences in the comparison.

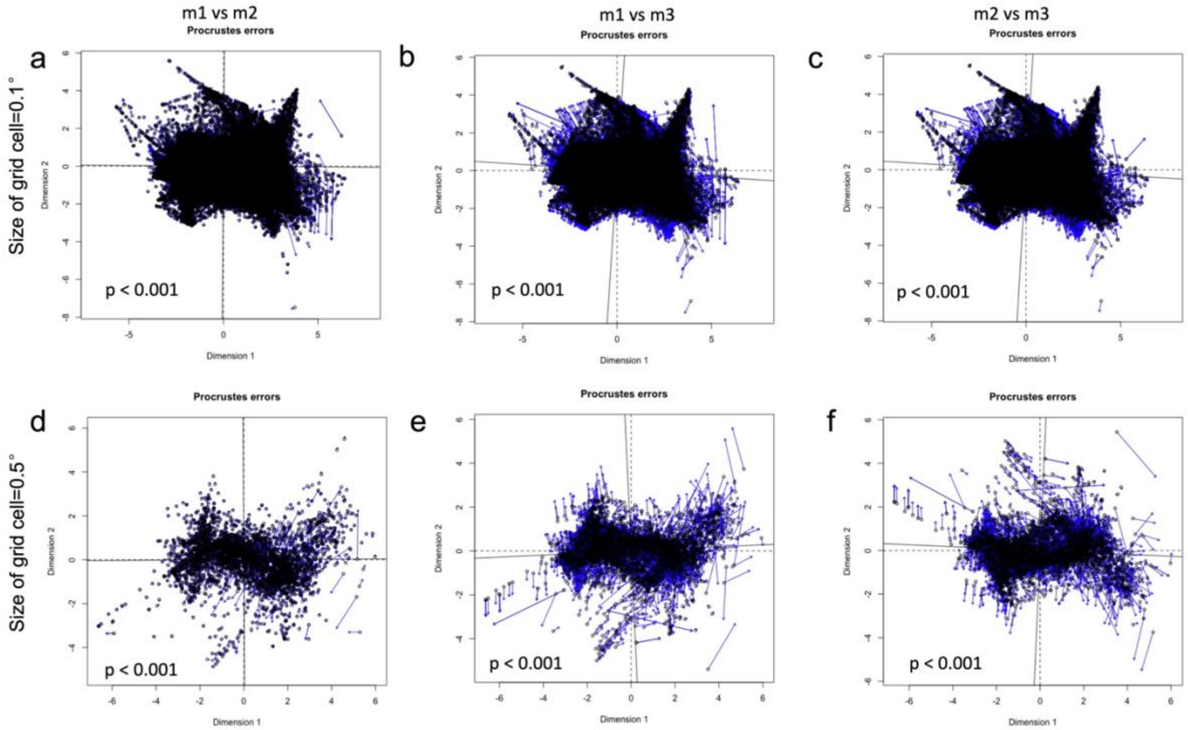

Figure S9. Procrustes tests for the comparison among three different gap-filling methods for the forest communities with different grid sizes. The grid sizes of 0.1° x 0.1° (a-c) and 0.5° x 0.5° (d-f) were used in our study. There are 49,805 and 3,798 forest communities for the 0.1° and 0.5° sizes, respectively. Three different methods were used to fill the missing trait gaps by applying (m1) the median of genus and family and phylogenetic relationship, (m2) median of genus and phylogenetic relationship and (m3) only the phylogenetic relationship. The loading values of the two main dimensions of the PCA tests were used for the comparisons (m1 vs m2 (a, d); m1 vs m3 (b, e) and m2 vs m3 (c, f). The visual indication of the degree of match between the two ordinations. The arrows point to their positions in the target ordination. The plot also shows the rotation between the two ordinations necessary to make them match as closely as possible. The p values (all p<0.001) indicate that there were no significant differences in the comparison.

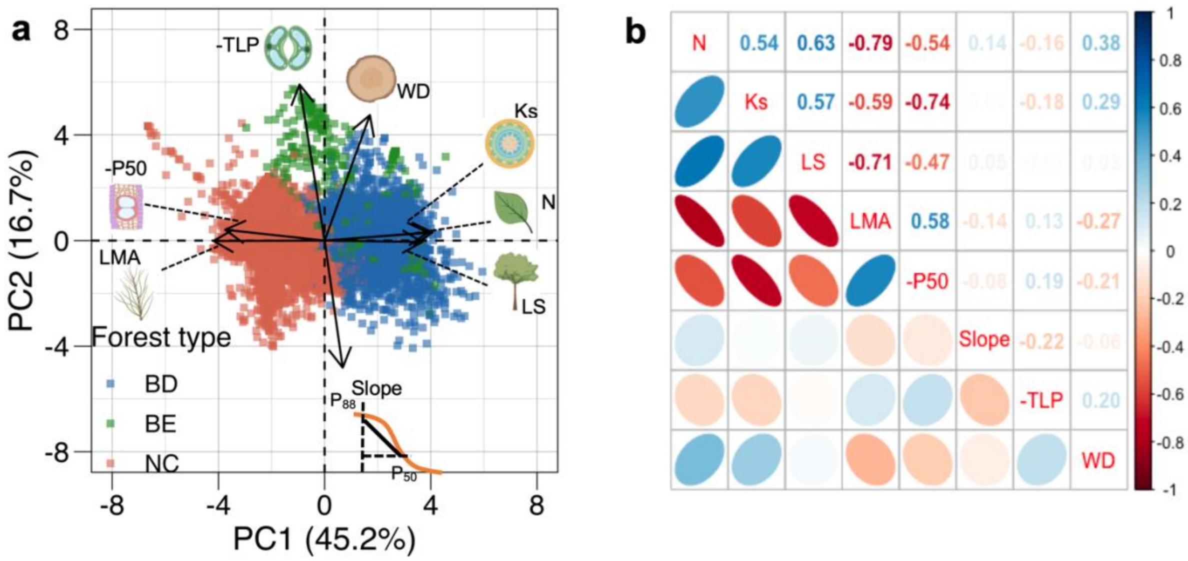

Figure S10. Sensitivity test for key functional strategies along the two dimensions for the forest communities larger than 2 plots. There are 10,071 forest communities used in this test. a. Biplot resulting from the principal component analysis (PCA) of eight functional traits at community level. The community-weighted mean trait value was calculated based on all species level trait values weighted by the proportions of basal area within a community. The dominant forest type was identified according to the relative proportion of dominant types representing more than 50% of grid basal area: BD (broadleaved deciduous, blue), BE (broadleaved evergreen, green) and NC (needleleaved conifer, red). b. Bar plots of the contribution of each trait (%) for the two main principal components (PC1 and PC2) at community level. b. Pearson correlation coefficient between the functional traits at community level are shown (all p values <0.001). Abbreviations of functional traits: N (leaf nitrogen content); Ks (maximum xylem conductivity per unit sapwood area); LS (leaf area to sapwood area ratio); LMA (leaf mass per area); P50 (xylem water potential at 50% loss of conductivity); Slope (slope for the embolism vulnerability curve between P50-P88); TLP (leaf turgor loss point) and WD (Wood density). The detailed traits and explanations are shown in Table 1. The elements in a are created in BioRender. Zhang, C. (2024) BioRender.com/u64o243.

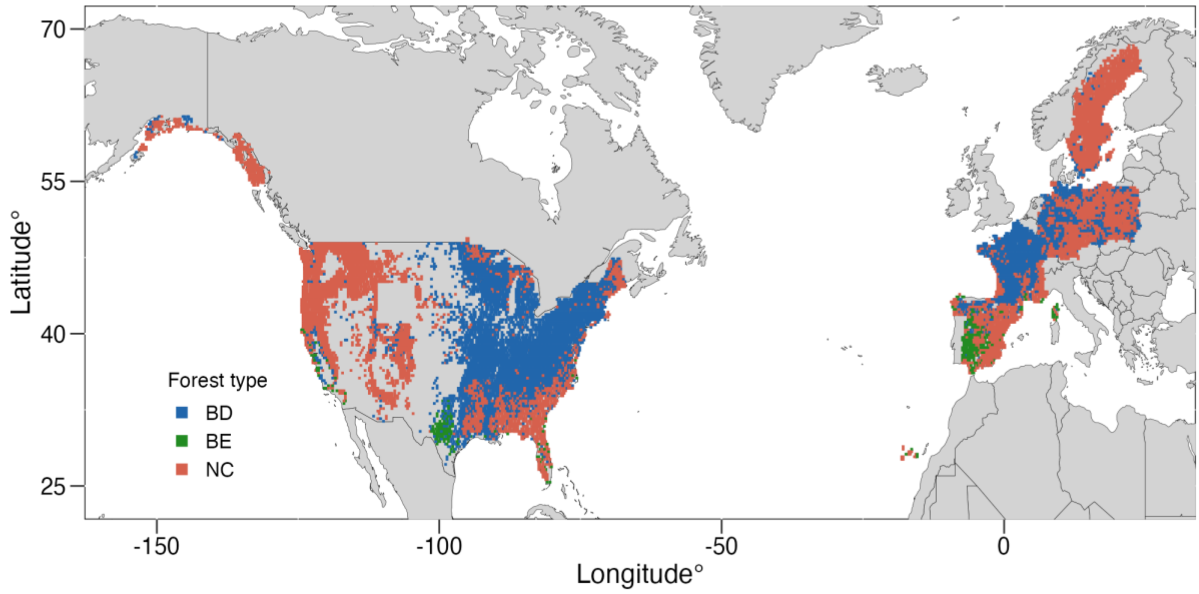

Figure S11. Geographic distribution of the dominant plant functional types across USA and Europe. The different colours indicate plant functional types (PFTs): broadleaved deciduous (BD, blue), broadleaved evergreen (BE, evergreen) and needleleaved conifer (NC, red). There are 12,452 forest communities used in the analysis, with 5,584 BD, 407 BE and 6,461 NC at the community level.

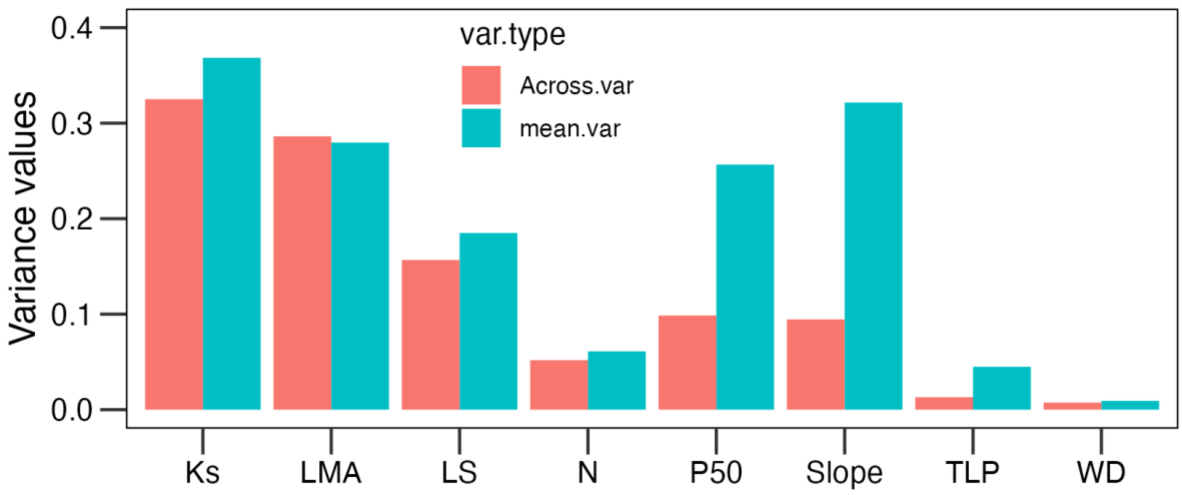

Figure S12. Comparing the mean trait variance within and across forest communities (red and light blue, respectively). There are 12,452 forest communities used in the analysis. The mean trait variance within forest communities was calculated as the average of the species-level trait variance within each community. The trait variance across the communities was calculated as the variance of each trait across all forest communities. Abbreviations of functional traits: N (leaf nitrogen content); Ks (maximum xylem conductivity per unit sapwood area); LS (leaf area to sapwood area ratio); LMA (leaf mass per area); P50 (xylem water potential at 50% loss of conductivity); Slope (slope for the embolism vulnerability curve between P50-P88); TLP (leaf turgor loss point) and WD (Wood density). The detailed traits and explanations are shown in Table 1.

Table S1. Forest inventory data used in our paper. The data described the regions, country, number of plots (n. plots), number of species (n. species), diameter threshold (D. threshold), the forest plot size and the mean census year of all forest plots.

Regions	Country	N. plots	N. species	D. threshold (mm)	Plot size (hectare)	Mean census year
North America	USA	59,624	475	127	0.067-0.45	2009.1
Europe	Spain	49,369	132	100	0.25	2005.3
	France	29,132	130	100	0.071	2012.0
	Germany	47,924	51	100	NA	2011.7
	Czech Republic	590	42	120	0.05	2009.2
	Poland	21,387	66	100	0.004-0.05	2009.7
	Sweden	13,505	24	100	0.031	2009.9

NA: The angle-count sampling technique does not have a fixed plot area.

Table S2. Percentage of traits missing for the woody species in our analyses. The traits were filled by median values of the genus and family separately.

Traits	Trait after initial species matching	Trait filled by genus	Trait filled by family
N	39%	12.4%	3.6%
Ks	76.9%	39.1%	18.2%
LS	83.1%	50.9%	25.8%
LMA	59.2%	24.8%	10.6%
-P50	67.2%	30.3%	12.7%
Slope	74.5%	39.6%	18.9%
-TLP	80.1%	43.8%	13.8%
WD	39.8%	11.6%	3.6%
