# Supplementary material for: Mapping multi-dimensional variability in water stress strategies across temperate forests
Source: Nat Commun. 2024 Oct 16;15:8909. doi: 10.1038/s41467-024-53160-1 (PMC11484845; doi:10.1038/s41467-024-53160-1)
Supplement: Supplementary file 3 — Description of Additional Supplementary Files [file 41467_2024_53160_MOESM3_ESM.pdf]

Description of Additional Supplementary Files

File Name: Supplementary Data 1

Description: The publication for the traits searched from Web of Science and Google Scholar.
