# Supplementary material for: Mapping multi-dimensional variability in water stress strategies across temperate forests
Source: Nat Commun. 2024 Oct 16;15:8909. doi: 10.1038/s41467-024-53160-1 (PMC11484845; doi:10.1038/s41467-024-53160-1)
Supplement: Supplementary file 5 — Reporting Summary [file 41467_2024_53160_MOESM5_ESM.pdf]

nature portfolio

Corresponding author(s): Daijun Liu

Last updated by author(s): Sep 13, 2024

Reporting Summary

Nature Portfolio wishes to improve the reproducibility of the work that we publish. This form provides structure for consistency and transparency in reporting. For further information on Nature Portfolio policies, see our Editorial Policies and the Editorial Policy Checklist.

Statistics

For all statistical analyses, confirm that the following items are present in the figure legend, table legend, main text, or Methods section.

n/a	Confirmed	
☐	☒	The exact sample size (*n*) for each experimental group/condition, given as a discrete number and unit of measurement
☐	☒	A statement on whether measurements were taken from distinct samples or whether the same sample was measured repeatedly
☐	☒	The statistical test(s) used AND whether they are one- or two-sided<br>*Only common tests should be described solely by name; describe more complex techniques in the Methods section.*
☐	☒	A description of all covariates tested
☐	☒	A description of any assumptions or corrections, such as tests of normality and adjustment for multiple comparisons
☐	☒	A full description of the statistical parameters including central tendency (e.g. means) or other basic estimates (e.g. regression coefficient) AND variation (e.g. standard deviation) or associated estimates of uncertainty (e.g. confidence intervals)
☐	☒	For null hypothesis testing, the test statistic (e.g. *F*, *t*, *r*) with confidence intervals, effect sizes, degrees of freedom and *P* value noted<br>*Give P values as exact values whenever suitable.*
☐	☒	For Bayesian analysis, information on the choice of priors and Markov chain Monte Carlo settings
☐	☒	For hierarchical and complex designs, identification of the appropriate level for tests and full reporting of outcomes
☐	☒	Estimates of effect sizes (e.g. Cohen's *d*, Pearson's *r*), indicating how they were calculated

Our web collection on statistics for biologists contains articles on many of the points above.

Software and code

Policy information about availability of computer code

Data collection	The trait data for all woody species across the USA and Europe can be found in the Zenodo repository (https://zenodo.org/records/13757078). All the community-level traits, PC scores and climate variables are available. The R code for the statistical analyses and generating the figures is also available.
Data analysis	All of the analyses in this study were conducted in R (version 4.2.3) (R Core Team 2023) and figures were produced by the ggplot2 R package (version 3.4.3).

For manuscripts utilizing custom algorithms or software that are central to the research but not yet described in published literature, software must be made available to editors and reviewers. We strongly encourage code deposition in a community repository (e.g. GitHub). See the Nature Portfolio guidelines for submitting code & software for further information.

Data

Policy information about availability of data

All manuscripts must include a data availability statement. This statement should provide the following information, where applicable:

- Accession codes, unique identifiers, or web links for publicly available datasets
- A description of any restrictions on data availability
- For clinical datasets or third party data, please ensure that the statement adheres to our policy

All the data are available in the Zenodo repository (https://zenodo.org/records/13757078).

Research involving human participants, their data, or biological material

Policy information about studies with human participants or human data. See also policy information about sex, gender (identity/presentation), and sexual orientation and race, ethnicity and racism.

Reporting on sex and gender	Not used in this study
Reporting on race, ethnicity, or other socially relevant groupings	Not used in this study
Population characteristics	Not used in this study
Recruitment	Not used in this study
Ethics oversight	Not used in this study

Note that full information on the approval of the study protocol must also be provided in the manuscript.

Field-specific reporting

Please select the one below that is the best fit for your research. If you are not sure, read the appropriate sections before making your selection.

☐ Life sciences ☐ Behavioural & social sciences ☒ Ecological, evolutionary & environmental sciences

For a reference copy of the document with all sections, see nature.com/documents/nr-reporting-summary-flat.pdf

Ecological, evolutionary & environmental sciences study design

All studies must disclose on these points even when the disclosure is negative.

Study description	We combined a large dataset of functional traits for woody plants with forest inventory plot data across regions of the USA (United States of America) and Europe (Spain, France, Germany, Poland, Czech Republic and Sweden). We considered eight continuous functional traits related to potential forest functional strategies to water stress (Table 1), concentrating on acquisitive-conservative, structural, stomatal and water storage strategies, as the information on rooting traits is very limited. We aggregated these forest plots into a grid level (0.25° grid cells) (here after community-level) to reduce stochasticity and then calculated community-weighted mean traits for each community separately.
Research sample	We used a total of 219,787 forest inventory plots across the USA and Europe in our analyses. We selected the living trees in each plot and included only those with a diameter at breast height larger than 12.7 cm across all the datasets. We aggregated inventory plots into 0.25° grid cells to dampen variation induced by the small sizes of plots and to provide a consistent spatial unit across all the countries.
Sampling strategy	We used grid-cell sizes of 0.1º and 0.5º, and applied the PCA to test community-level trait variation and trade-offs among the traits. We also conducted the PCA tests of the eight community-level traits using different methods to fill the missing gaps by applying (a) the median of genus and family and phylogenetic relationship, (b) median of genus and phylogenetic relationship and (c) only the phylogenetic relationship.
Data collection	We used a total of 219,787 forest inventory plots across the USA and Europe in our analyses. We selected the living trees in each plot and included only those with a diameter at breast height larger than 12.7 cm across all the datasets. All of the plot data were provided by coauthors.
Timing and spatial scale	To keep the forest inventory data temporally consistent between countries, censuses closest to the year of 2010 were selected since the majority of the forest inventory data were available at that period. We used forest inventory data from the USA and six countries in Europe (Spain, France, Germany, Czech Republic, Poland and Sweden) (Table. S1).
Data exclusions	All of the data were included into the analysis.
Reproducibility	All the data for the analyses and code for making figures1-4 are available for readers.
Randomization	The randomization was not relevant to the work.
Blinding	Blinding was not applicable in this submission process.

Did the study involve field work? ☐ Yes ☒ No

Reporting for specific materials, systems and methods

We require information from authors about some types of materials, experimental systems and methods used in many studies. Here, indicate whether each material, system or method listed is relevant to your study. If you are not sure if a list item applies to your research, read the appropriate section before selecting a response.

Materials & experimental systems

n/a	Involved in the study
☒	☐ Antibodies
☒	☐ Eukaryotic cell lines
☒	☐ Palaeontology and archaeology
☒	☐ Animals and other organisms
☒	☐ Clinical data
☒	☐ Dual use research of concern
☒	☐ Plants

Methods

n/a	Involved in the study
☒	☐ ChIP-seq
☒	☐ Flow cytometry
☒	☐ MRI-based neuroimaging
